# Comparison of predicted susceptibility between genotype and virtual phenotype HIV drug resistance interpretation systems among treatment-naive HIV-infected patients in Asia: TASER-M cohort analysis

**DOI:** 10.1186/1756-0500-5-582

**Published:** 2012-10-24

**Authors:** Awachana Jiamsakul, Rami Kantor, Patrick CK Li, Sunee Sirivichayakul, Thira Sirisanthana, Pacharee Kantipong, Christopher KC Lee, Adeeba Kamarulzaman, Winai Ratanasuwan, Rossana Ditangco, Thida Singtoroj, Somnuek Sungkanuparph

**Affiliations:** 1 The Kirby Institute, University of New South Wales, Sydney, Australia; 2 Division of Infectious Diseases, Brown University Alpert Medical School, Rhode Island, USA; 3 Department of Medicine, Queen Elizabeth Hospital, Hong Kong, China; 4 Faculty of Medicine, Chulalongkorn University and HIV-NAT/Thai Red Cross AIDS Research Centre, Bangkok, Thailand; 5 Research Institute for Health Sciences, Chiang Mai University, Chiang Mai, Thailand; 6 Chiangrai Prachanukroh Regional Hospital, Chiang Rai, Thailand; 7 Hospital Sungai Buloh, Sungai Buloh, Malaysia; 8 University of Malaya Medical Center, Kuala Lumpur, Malaysia; 9 Siriraj Hospital, Mahidol University, Bangkok, Thailand; 10 Research Institute for Tropical Medicine, Manila, Philippines; 11 TREAT Asia, amfAR - The Foundation for AIDS Research, Bangkok, Thailand; 12 Faculty of Medicine Ramathibodi Hospital, Mahidol University, Bangkok, Thailand

**Keywords:** Asia, HIV, Resistance, Interpretation, Algorithm

## Abstract

**Background:**

Accurate interpretation of HIV drug resistance (HIVDR) testing is challenging, yet important for patient care. We compared genotyping interpretation, based on the Stanford University HIV Drug Resistance Database (Stanford HIVdb), and virtual phenotyping, based on the Janssen Diagnostics BVBA’s vircoTYPE™ HIV-1, and investigated their level of agreement in antiretroviral (ARV) naive patients in Asia, where non-B subtypes predominate.

**Methods:**

Sequences from 1301 ARV-naive patients enrolled in the TREAT Asia Studies to Evaluate Resistance – Monitoring Study (TASER-M) were analysed by both interpreting systems. Interpretations from both Stanford HIVdb and vircoTYPE™ HIV-1 were initially grouped into 2 levels: susceptible and non-susceptible. Discrepancy was defined as a discordant result between the susceptible and non-susceptible interpretations from the two systems for the same ARV. Further analysis was performed when interpretations from both systems were categorised into 3 levels: susceptible, intermediate and resistant; whereby discrepancies could be categorised as major discrepancies and minor discrepancies. Major discrepancy was defined as having a susceptible result from one system and resistant from the other. Minor discrepancy corresponded to having an intermediate interpretation in one system, with a susceptible or resistant result in the other. The level of agreement was analysed using the prevalence adjusted bias adjusted kappa (PABAK).

**Results:**

Overall, the agreement was high, with each ARV being in “almost perfect agreement”, using Landis and Koch’s categorisation. Highest discordance was observed for efavirenz (75/1301, 5.8%), all arising from susceptible Stanford HIVdb versus non-susceptible vircoTYPE™ HIV-1 predictions. Protease Inhibitors had highest level of concordance with PABAKs all above 0.99, followed by Nucleoside Reverse Transcriptase Inhibitors with PABAKs above 0.97 and non-NRTIs with the lowest PABAK of 0.88. The 68/75 patients with discordant efavirenz results harboured the V179D/E mutations compared to 7/1226 with no efavirenz discrepancy (p-value <0.001). In the 3-level comparison, all but one of the discrepancies was minor.

**Conclusions:**

The two systems agreed well with lowest concordance observed for efavirenz. When interpreting HIVDR, especially in non-B subtypes, clinical correlation is crucial, in particular when efavirenz resistance is interpreted based on V179D/E.

## Background

In recent years, developing countries have experienced a rapid expansion of antiretroviral drugs (ARVs) in the treatment of HIV-1 infection [1]. Treatment-naive HIV-infected patients may harbour drug-resistance-associated mutations (RAMs) prior to their initial treatment through infection from pre-treated patients. The prevalence of RAMs in treatment-naive patients in resource limited settings was found to vary between different settings [2-5], mainly below 10%. This implies that drug resistance monitoring prior to treatment may aid the selection of appropriate ARVs.

The aim of the Therapeutics, Research, Education and AIDS Training in Asia (TREAT Asia) Studies to Evaluate Resistance – Monitoring Study (TASER–M) is to monitor HIV-1 drug resistance (HIVDR) and its effects in HIV-infected patients in Asia. The TASER-M cohort consists mainly of ARV-naive Asian individuals infected with HIV-1 AE circulating recombinant form (CRF01_AE) [6]. This is in contrast to developed countries where HIV-1 subtype B predominates. HIV-1 subtypes differ in their gene sequences and this may influence susceptibility to ARVs [7,8]. Some non-B subtypes have natural polymorphisms at resistance associated positions [9], leading to possible misinterpretation of drug susceptibility.

There are several software-based drug resistance interpretation systems currently available, which are used in patient care to design subsequent regimens. The two systems adopted in TASER-M are (i): the Stanford University HIV Drug Resistance Database (Stanford HIVdb) which has a publicly available tool that provides genotypic analysis and interpretation via web-based sequence submission. The Stanford HIVdb uses a rules based system in which all mutation scores are added to derive a level of predicted viral resistance to each ARV. These rules are pre-determined based on research findings of published studies [10,11]; (ii) Janssen Diagnostics BVBA’s vircoTYPE™ HIV-1 which is a proprietary software that provides a predicted or ‘virtual’ phenotype of a sequence, based on a large genotype-phenotype database. The tool provides a calculated fold change (FC) predicted phenotype of the patient’s genotype, compared to a reference sequence, derived from linear regression modelling [12,13]. The Stanford HIVdb and vircoTYPE™ HIV-1 utilise consensus B and HXB2, respectively, as the reference strains [10,11,13,14].

Because different interpretation algorithms use different rules to predict drug susceptibility, results may differ. Studies have shown that differences do exist with varying degree of discordances [15-21], and the proportion of discordances for each ARV could be subtype dependent [22,23].

The objective of this study was to determine the yet unstudied level of agreement between genotype and virtual phenotype from the Stanford HIVdb and vircoTYPE™ HIV-1 resistance interpretation in the TASER-M cohort, where non-B subtypes predominate.

## Methods

TASER-M recruitment began in 2007, with a total of 12 participating sites from Thailand, Hong Kong, Malaysia, Philippines and Indonesia. The TASER-M cohort comprises of 95% treatment naive patients at time of enrolment with pre-treatment sequences contributing to more than 90% of total available sequences. Ethics approvals were obtained from the University of New South Wales Ethics Committee and institutional review boards at the participating clinical sites. Informed consent was obtained from participants prior to enrolment. ARV treatment-naive patients were required to have viral load and resistance testing performed prior to initiation of ARVs and at yearly intervals post initiation [24]. Genotyping was performed in local TAQAS [25] laboratories.

Sequences were included in this analysis if they were pre-treatment sequences derived from HIV-infected patients enrolled as treatment-naive in the TASER-M cohort. FASTA sequence files were analysed by both the Stanford HIVdb and vircoTYPE™ HIV-1. FASTA files were submitted to the Stanford HIVdb (Version 6.0.11) through Sierra – The Stanford HIV Web Service (Version 1.0) for genotypic interpretation. Subtyping was analysed using REGA HIV-1 Subtyping Tool - Version 2.0.

For virtual phenotyping, FASTA files were submitted to Janssen Diagnostics. The vircoTYPE™ HIV-1 (VPT 4.3.01) results were then transferred to Advanced Biological Laboratories (ABL) TherapyEdge 3.9.2 web based software, and retrieved directly from TherapyEdge 3.9.2. The interpretation of the FC is made in reference to the clinical cut-offs (CCOs) or biological cut-offs (BCOs) [26,27]. If the CCOs are used, the resistance interpretations are categorised as maximal response if the FC is less than the lower CCO (CCO1), reduced response if the FC is between CCO1 and upper CCO (CCO2), and minimal response if the FC is greater than CCO2. For ARVs utilising the BCO, the interpretations are either susceptible if the FC is below the BCO, or resistant if the FC is above the BCO [14]. For lamivudine and etravirine, a combination of both BCO and CCO are used in vircoTYPE™ HIV-1 predictions. The lower CCO (CCO1) is replaced by the BCO, resulting in two cut-off points: BCO and CCO2. If the FC value is below the BCO, the interpretation will be susceptible. If the FC is between the BCO and CCO2, the result will be classified as reduced response, whilst minimal response is the resulting interpretation when FC is greater than CCO2 for these three drugs.

### Two level interpretations

A total of 15 ARV interpretations (7 Nucleoside Reverse Transcriptase Inhibitors (NRTIs), 3 non-NRTIs (NNRTIs) and 5 Protease Inhibitors (PIs)) and were compared. The Stanford HIVdb provides 5 levels of susceptibility prediction for each ARV (susceptible, potential low-level resistance, low-level resistance, intermediate resistance and high-level resistance). The vircoTYPE™ HIV-1 utilises either 2 or 3-level resistance prediction for any given ARV, depending on the cut-offs used.

To be able to compare both systems effectively, the 5-level interpretations from the Stanford HIVdb needed to be combined in order to match the levels of interpretation provided by vircoTYPE™ HIV-1. For comparison of all ARVs, 2-level comparisons were performed to enable the inclusion of ARVs where virtual phenotyping only provided predictions at 2 levels. That is, the 3-level interpretations available from the Stanford’s Sierra database (susceptible, intermediate and resistant), initially grouped from the 5-level interpretation, were combined further into susceptible (susceptible) and non-susceptible (intermediate and resistant). The vircoTYPE™ HIV-1’s susceptible and maximal response were categorised as susceptible whilst reduced response, minimal response and resistant were grouped as non-susceptible. Discrepancy was defined as a discordant drug resistant interpretation between the Stanford HIVdb and the vircoTYPE™ HIV-1 report, using the 2-level interpretations, i.e. susceptible versus non-susceptible, and vice versa.

The ARV that exhibited the highest discordances was chosen for further investigation into the mutation pattern and predicted FC values associated with the discrepancies. The mutations were analysed using the Stanford’s HIV Drug Resistance Mutations by Drug Class (November 6, 2009) [28] (Stanford HIVDR list) only, as vircoTYPE™ HIV-1’s list of relevant mutations was not available. Differences in FC values were analysed by comparing the median FC between the discordant and non-discordant groups.

### Three level interpretations

To evaluate whether the discrepancies were major or minor in nature, a sub comparison was also made using 3-level susceptibility predictions. In this comparison however, efavirenz, nevirapine and emtricitabine were excluded as they did not have 3 levels of virtual phenotype interpretations available. As a consequence, the available prediction output for all other ARVs were susceptible, maximal response, reduced response and minimal response. The susceptible category here belongs to lamivudine and etravirine as a result of the BCO being used as the lower cut off point, instead of the CCO1, thus replacing “maximal response” with “susceptible” interpretation. The vircoTYPE™ HIV-1’s susceptible and maximal response were then categorised as susceptible, while reduced response was intermediate, and minimal response was defined as resistant. The interpretations were then compared to the 3-level Stanford HIVdb results. Major discrepancy was defined as having a susceptible result from one system and resistant from the other. Minor discrepancy corresponded to having an intermediate interpretation in one system, with a susceptible or resistant result in the other.

### Statistical analysis

To take into account agreement due to chance, the prevalence adjusted bias adjusted kappa coefficient (PABAK) was used to analyse the level of agreement between the two interpretation systems [29]. The interpretation of PABAK was made based on Landis and Koch’s [30] categorisation: 0 to 0.20 slight agreement; 0.21 to 0.40 fair agreement; 0.41 to 0.60 moderate agreement; 0.61 to 0.80 substantial agreement; and 0.81 to 1.00 almost perfect agreement.

Median values were analysed using Wilcoxon rank sum test. Categorical data was analysed using Chi-square, Fisher’s exact test or McNemar’s test.

Analyses were performed using SAS software version 9.2 (SAS Institute Inc., Cary, NC, USA) and STATA software version 10.1 (STATA Corp., College Station, TX, USA). Ordinal scale PABAK (PABAK-OS) for 3-level interpretation was analysed using PABAK-OS web-based tool [31].

## Results

### Demographics

A total of 1301 eligible naive patients consented between 2007 and September 2010 were included. Out of the 1301 baseline sequence files analysed, most were from male patients (66%) with a median age of 36 years (IQR 31–44). The main ethnicity was Thai (71%) followed by Chinese (20%). Heterosexual contact was the predominant mode of exposure (72%). The median pre-treatment CD4 and RNA counts were 100 cells/μL (IQR 34–199) and 100,000 copies/mL (IQR 41,345 – 230,000), respectively. The main subtype was CRF01_AE (78%). Subtype B accounted for 15% and other non-B subtypes were 7%.

### Two level interpretations

**Figure 1:**
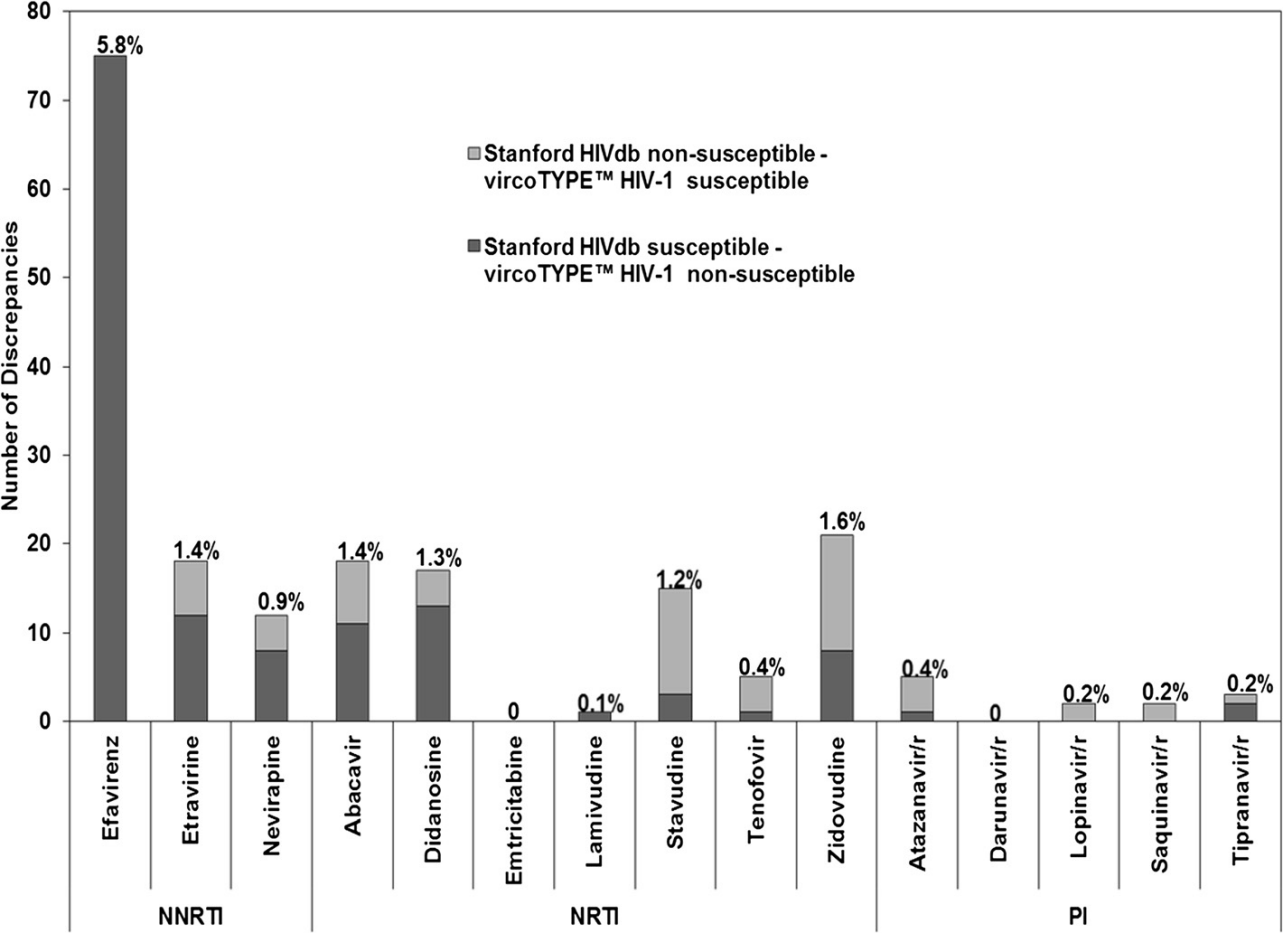
Number and percentage of discordant drug resistance interpretations for each ARV – 2-level interpretation.

The number of discrepant results, using the 2-level interpretation method, between Stanford HIVdb and the vircoTYPE™ HIV-1 for each ARV are illustrated in Figure 1. The number of discrepancies ranged from 0 to 75. The ARV with the highest number of discordances was efavirenz (75/1301, 5.8%), followed by zidovudine (21/1301, 1.6%) and etravirine and abacavir (18/1301, 1.4% each). All of the 75 discrepancies for efavirenz arose from susceptible interpretation from the Stanford HIVdb versus non-susceptible interpretation from vircoTYPE™ HIV-1. The observed efavirenz agreement was 94% with 1199 patients being susceptible and 27 non-susceptible. For zidovudine, the observed agreement was 98% with 1274 susceptible and 6 non susceptible patients. Emtricitabine and darunavir/r both had 100% agreement with no discrepancy, however 1293/1301 agreements for emtricitabine were from susceptible prediction and 8/1301 were non-susceptible. For darunavir/r, 100% of the agreements were susceptible from both systems.

The PABAK values (Table 1) ranged from 0.88 to 1.00. The lowest PABAK (0.88) was observed for efavirenz. This is consistent with efavirenz having the lowest number of concordances. The PABAK for etravirine, abacavir, didanosine and zidovudine was second lowest at 0.97. For the two drugs with no discrepancy, the corresponding PABAK for both drugs was 1.00 with 95% confidence interval (95%CI) of (1.00-1.00). Overall, PIs had highest level of concordance with individual PABAK values all above 0.99, followed by NRTIs with PABAKs above 0.97 and NNRTIs with lowest PABAK of 0.88. Using Landis and Koch’s categorisation, the agreement between the two interpreting systems for all ARVs considered could be classified as “almost perfect agreement” since the PABAK values all lie within the 0.81-1.00 interval.

**Table 1:** PABAK values for each ARV – 2-level interpretation

**Drug class**	**Drug**	**Proportion observed agreement**	**PABAK**	**95%CI of PABAK**
**NNRTI**	Efavirenz	0.94	0.88	(0.86 - 0.91)
	Etravirine	0.99	0.97	(0.96 - 0.99)
	Nevirapine	0.99	0.98	(0.97 - 0.99)
**NRTI**	Abacavir	0.99	0.97	(0.96 - 0.99)
	Didanosine	0.99	0.97	(0.96 - 0.99)
	Emtricitabine	1.00	1.00	(1.00 -1.00)
	Lamivudine	1.00	1.00	(1.00 -1.00)
	Stavudine	0.99	0.98	(0.97 - 0.99)
	Tenofovir	1.00	0.99	(0.99 - 1.00)
	Zidovudine	0.98	0.97	(0.95 - 0.98)
**PI**	Atazanavir/r	1.00	0.99	(0.99 - 1.00)
	Darunavir/r	1.00	1.00	(1.00 - 1.00)
	Lopinavir/r	1.00	1.00	(0.99 - 1.00)
	Saquinavir/r	1.00	1.00	(0.99 - 1.00)
	Tipranavir/r	1.00	1.00	(0.99 - 1.00)

As efavirenz had significantly higher number of discordances compared to other ARVs (p-value<0.001 for all comparisons) and the lowest PABAK, it was chosen for further investigation. The relevant mutations were extracted from the Stanford HIVdb and compared against the Stanford HIVDR list only. 68/75 (91%) patients with discrepant efavirenz results harboured the V179D/E mutations (V179D (83%), V179E (17%)) compared to only 7/1226 (0.6%) of patients with no efavirenz discrepancy (p-value <0.001). Apart from V179D/E, these 68 patients did not present any other efavirenz RAMs. The other 7/75 (9%) patients who had the discrepancy did not harbour any efavirenz-associated mutations. These 7 discrepant patients may harbour other RAMs that were found to significantly affect susceptibility to efavirenz by the vircoTYPE™ HIV-1’s algorithm, which were not present in the Stanford HIVDR list. Of note, T39K, K43S, K122E, D123S, D177E, Q207A and R211S were the most common RT-mutations (86%) found in these 7 patients with respect to the reference wildtype virus. However no common RT-mutation was identified across all 7 patients. Out of the 75 patients with efavirenz discrepancy, 79% were non-B subtype compared to 85% in patients showing concordance (p=0.135). Among the 1226 patients with no discrepancy, the efavirenz-associated mutations were A98G (0.3%), K103N (0.8%), K103S (0.2%), V106M (0.2%), V179D (0.6%), Y181C (0.6%), G190A (0.3%), G190E (0.1%), P225H (0.2%), F227C (0.1%) and K238T (0.1%). RAMs found in discrepant patients for other NNRTIs were: etravirine (18 discordances): V179D (38.9%), V179E (11.1%), G190A (5.6%) and G190E (5.6%); nevirapine (12 discordances) V179D (75%); V179E (8.3%) and F227C (8.3%). Although G190A/E and F227 were found in a proportion of these patients, a similar pattern to efavirenz could be seen whereby discrepant patients with V179D/E did not harbour any additional NNRTI RAMs.

In vircoTYPE™ HIV-1, the BCO was used to determine the level of susceptibility of efavirenz. Therefore only one cut-off point (BCO = 3.3) and two interpretations were available. There was no discrepancy found in patients with FC less than the assigned BCO of 3.3 for efavirenz. That is, all patients with the discrepancies had reported FC greater than 3.3, which resulted in a non-susceptible or resistant virtual phenotype interpretation.

In light of 100% efavirenz discrepant patients possessing non-susceptible virtual phenotype results, median FC values were compared between the discordant and concordant group for those with non-susceptible vircoTYPE™ HIV-1 interpretation only. This is because by restricting the comparison to those that resulted in non-susceptible interpretation (FC>3.3), it was possible to make direct comparison without diluting the results with the low FC values whereby no discrepancy was found. There was a total of 102 patients with FC above the BCO of 3.3 for efavirenz. Out of these 102 patients, 27 had no discrepant result, with median FC of 31.90 and inter quartile range (IQR) (7.10-87.10). The median FC for those with discrepancies was 5.10 (p-value <0.001; IQR 4.30-6.10). This suggests that for patients with non-susceptible virtual phenotype results for efavirenz, the group with the discordances had a median FC that is lower and nearer to the cut-off point than those without discordances.

### Three level interpretations

**Figure 2:**
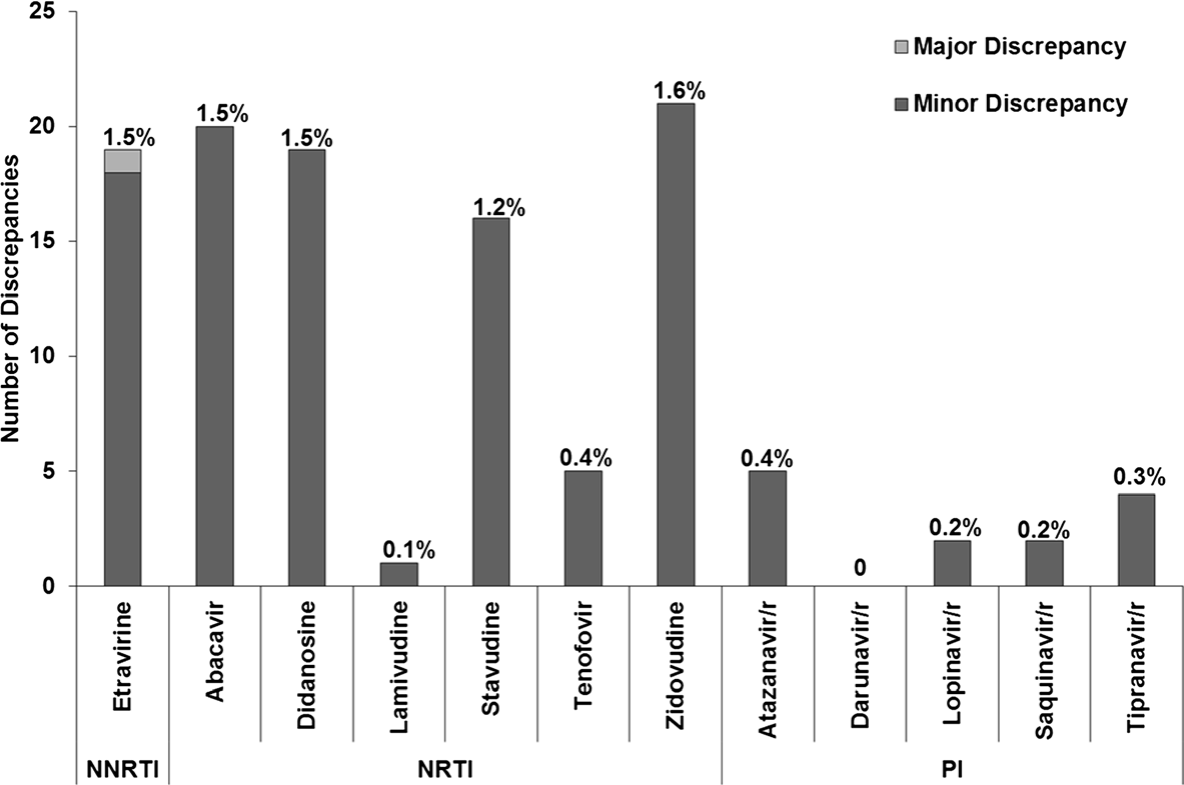
Number and percentage of major and minor discrepancies for each ARV– 3-level interpretation, excluding efavirenz, nevirapine and emtricitabine.

Interpretations were regrouped into susceptible, intermediate and resistant in order to determine the number of minor and major discrepancies. Efavirenz, emtricitabine and nevirapine were excluded. Figure 2 and Table 2 indicate that all of the discordances, but one, were classified as minor discrepancies. The proportion of exact agreement ranged from 98% to 100%. As with the two level interpretations, darunavir/r had 100% agreement with no discrepancy.

**Table 2:** PABAK – OS – 3-level interpretation

**Drug Class**	**Drug**	**Proportion observed agreement**	**Minor discrepancy**	**Major discrepancy**	**PABAK-OS**	**95%CI of PABAK-OS**
**NNRTI**	Etravirine	0.99	18	1	0.98	(0.96 - 1.01)
**NRTI**	Abacavir	0.98	20	0	0.98	(0.96 - 1.01)
	Didanosine	0.99	19	0	0.98	(0.96 - 1.01)
	Lamivudine	1.00	1	0	1.00	(0.97 - 1.03)
	Stavudine	0.99	16	0	0.99	(0.96 - 1.01)
	Tenofovir	1.00	5	0	1.00	(0.97 - 1.02)
	Zidovudine	0.98	21	0	0.98	(0.95 - 1.01)
**PI**	Atazanavir/r	1.00	5	0	1.00	(0.97 - 1.02)
	Darunavir/r	1.00	0	0	1.00	(0.97 - 1.03)
	Lopinavir/r	1.00	2	0	1.00	(0.97 - 1.03)
	Saquinavir/r	1.00	2	0	1.00	(0.97 - 1.03)
	Tipranavir/r	1.00	4	0	1.00	(0.97 - 1.02)

The results of Table 2 suggest that almost all of the discrepancies that exist between Stanford HIVdb and vircoTYPE™ HIV-1 were minor in nature. That is, one system produced an intermediate interpretation while the other produced either a susceptible or resistant interpretation. Etravirine was the only ARV that possessed a major discrepancy where the Stanford HIVdb’s interpretation was susceptible and vircoTYPE™ HIV-1 was resistant. Without the inclusion of efavirenz, the number of minor discrepancies in Table 2 ranged from 0 to 21. By comparing the 2 and 3-level discrepancies, it was found that the total number of discrepancies from the 3-level comparison was slightly greater than that from the 2-level comparison for a number of ARVs. For example, abacavir had 20 and 18 discrepancies in the 3 and 2-level comparisons, respectively. This is due to the fact that the differences between intermediate and resistant interpretations would not have been captured in the 2-level categorisation, as both interpretations were grouped as non-susceptible. However, the total discrepancies for each ARV between the 2 and 3-level groupings differed by a maximum of 2, and the PABAK and PABAK-OS agreement interpretations remained unchanged, therefore the simplification of the interpretations into two categories did not greatly alter the discordance outcome.

## Discussion

The overall results suggest that both the Stanford HIVdb and the vircoTYPE™ HIV-1 provide comparable drug resistance interpretations. In the 2-level comparison, efavirenz showed the highest number of disagreements, all of which were Stanford HIVdb susceptible - vircoTYPE™ HIV-1 resistant pairs. The PABAK values for all ARVs considered remained in the “almost perfect agreement” category. The results from the 2-level comparison suggest that the lowest level of agreement belongs to the NNRTI drug class. This is in contrast to previous studies [15-19] where NRTIs were found to have lowest concordances. Of note, the majority of the patients in this cohort harboured CRF01_AE subtype virus, whereas B subtype was predominant in preceding studies [16,17,19]. Studies comparing non-B sequences reported highest discordances in either PI or NRTI drug class [20,21].

The RAMs associated with efavirenz discrepancy were V179D/E. These are likely to be natural polymorphisms in HIV-1 RT. V179D/E are often found in treatment-naive individuals infected with non-B subtypes [6,32,33]. V179D is associated with low-level resistance to NNRTIs and is considered to have no significant effect on the efficacy of NNRTI–containing regimen. The 5-level Stanford HIVdb predicted “potential low-level resistance” in all sequences with discordant efavirenz interpretation containing V179D/E. A combination of V106I and V179D has been shown to confer significant resistance to efavirenz [34]. Among TASER-M patients, only 1 had a combination of V106I and V179D. V179D also confers significant resistance to NNRTIs when presented together with K103R [35]. Only 2 patients had a combination of K103R and V179D.

Since the mutations were extracted and compared against the Stanford HIVDR list only, it should be noted that the RAMs found to be associated with the discrepancies are those based on the Stanford HIVDR list alone. This is one limitation of the study. The likely presence of additional mutations in vircoTYPE™ HIV-1 coupled with the differences in mutation weights or scores are the most likely causes of the discrepancies. Therefore, the significance of V179D/E found in this study should not be interpreted as the cause of the differences between the two interpreting systems, but merely reflects the mutation pattern based on the Stanford HIVDR list. However, it has been reported that natural polymorphisms found in non-B subtype have contributed to the majority of the discordances, although this was mainly associated with the protease region of the sequence [20].

The 3-level comparison showed that most of the discrepancies found were minor in nature. These may have less clinical importance than the major discordances. The availability of only two virtual phenotype predictions for efavirenz, nevirapine and emtricitabine, another limitation of this study, did now allow for further break down of discordant groups. As such, discrepancies between intermediate and resistant categorisation for these three drugs could not be captured. Nevertheless, the minor discrepancies illustrated in this study suggest that the results did not differ greatly from one system to the other.

The differences in median FC between those with and without efavirenz discrepancies suggest that one should take into consideration the actual predicted FC values in addition to the resistance interpretation when evaluating efavirenz resistance. Although discrepancies only occurred in sequences with resistant interpretation, those who were resistant with lower FC tended to result in discordant interpretation compared to those with high FC. Additionally, by simplifying resistance calls into categories, the true value of predicted phenotype is diminished. In the Stanford HIVdb, the 3 or 5-level classification should also be interpreted together with the resistance scores in order to take into account the variation of susceptibility within each classification. It would be of interest to re investigate when the CCOs become available for efavirenz, nevirapine and emtricitabine. It is likely that the FC values associated with efavirenz discrepancies would lie in the middle region between CCO1 and CCO2, resulting in minor discrepancies.

## Conclusions

This study has shown high concordance between the Stanford HIVdb and vircoTYPE™ HIV-1 in naive patients. The differences in the proportions of discordances from different patient groups reported in previous studies [20,22,23] and the higher efavirenz discrepancies compared to all other ARVs analysed indicate that more data is required to enable the algorithms to adapt appropriately to different sequence variations. When interpreting HIVDR, especially in non-B subtypes, careful mutation-specific, interpretation of genotyping and virtual phenotyping results are recommended to identify potential discordances.

## Competing interests

The authors declare that they have no competing interests.

## Authors’ contributions

AJ performed data manipulation, analysis plan, statistical analysis, interpretation of results and drafted the manuscript. SS originated the study concept, participated in the analysis plan, data collection, helped interpret results and edited the manuscript. RK participated in the analysis plan, helped interpret results and edited the manuscript. PL, SS, TS, PK, CL, AK, WR, RD, TS performed data collection and revised the final manuscript. All authors read and approved the final manuscript.

## Members of the TASER study include

· PCK Li† and MP Lee, Queen Elizabeth Hospital and KH Wong, Integrated Treatment Centre, Hong Kong, China;

· N Kumarasamy and S Saghayam, YRG Centre for AIDS Research and Education, Chennai, India;

· S Pujari and K Joshi, Institute of Infectious Diseases, Pune, India;

· TP Merati‡ and F Yuliana, Faculty of Medicine, Udayana University & Sanglah Hospital, Bali, Indonesia;

· A Kamarulzaman and LY Ong, University Malaya Medical Center, Kuala Lumpur, Malaysia;

· C KC Lee and B HL Sim, Hospital Sungai Buloh, Sungai Buloh, Malaysia;

· M Mustafa and N Nordin, Hospital Raja Perempuan Zainab II, Kota Bharu, Malaysia;

· R Ditangco and RO Bantique, Research Institute for Tropical Medicine, Manila, Philippines;

· YMA Chen, YJ Chen and YT Lin, Taipei Veterans General Hospital and AIDS Prevention and Research Centre, National Yang-Ming University, Taipei, Taiwan;

· P Kantipong and P Kambua, Chiang Rai Regional Hospital, Chiang Rai, Thailand;

· P Phanuphak and S Sirivichayakul, HIV-NAT/Thai Red Cross AIDS Research Centre, Bangkok, Thailand;

· W Ratanasuwan and R Sriondee, Faculty of Medicine, Siriraj Hospital, Mahidol University, Bangkok, Thailand;

· T Sirisanthana and J Praparattanapan, Research Institute for Health Sciences, Chiang Mai University, Chiang Mai, Thailand;

· S Sungkanuparph, S Kiertiburanakul, and L Chumla, Faculty of Medicine, Ramathibodi Hospital, Mahidol University, Bangkok, Thailand;

· R Kantor, Brown University, Rhode Island, U.S.A.;

· AH Sohn, N Durier and T Singtoroj, TREAT Asia, amfAR -- The Foundation for AIDS Research, Bangkok, Thailand;

· DA Cooper, MG Law, and A Jiamsakul, The Kirby Institute, University of New South Wales, Sydney, Australia.

† Steering Committee Chair, ‡ Co-Chair

The content of this publication is solely the responsibility of the authors and does not necessarily represent the official views of any of the institutions mentioned above.
